# Effect of Freeze Drying and Simulated Gastrointestinal Digestion on Phenolic Metabolites and Antioxidant Property of the Natal Plum (*Carissa macrocarpa*)

**DOI:** 10.3390/foods10061420

**Published:** 2021-06-18

**Authors:** Faith Seke, Vimbainashe E. Manhivi, Tinotenda Shoko, Retha M. Slabbert, Yasmina Sultanbawa, Dharini Sivakumar

**Affiliations:** 1 Department of Horticulture, Tshwane University of Technology, Pretoria West 0001, South Africa; fayeesk@gmail.com (F.S.); SlabbertMM@tut.ac.za (R.M.S.); 2 Phytochemical Food Network Group, Department of Crop Sciences, Tshwane University of Technology, Pretoria West 0001, South Africa; ManhiviVE@tut.ac.za (V.E.M.); ShokoT@tut.ac.za (T.S.); 3 Australian Research Council Industrial Transformation Training Centre for Uniquely Australian Foods, Queensland Alliance for Agriculture and Food Innovation, Center for Food Science and Nutrition, The University of Queensland, St Lucia, QLD 4069, Australia; y.sultanbawa@uq.edu.au

**Keywords:** functional powder, phytochemicals, antioxidants, bioaccessibility, cyanidin-3-O-β-sambubioside

## Abstract

Natal plums (*Carissa macrocarpa*) are a natural source of bioactive compounds, particularly anthocyanins, and can be consumed as a snack. This study characterized the impact of freeze drying and *in vitro* gastrointestinal digestion on the phenolic profile, antioxidant capacity, and α-glucosidase activity of the Natal plum (*Carissa macrocarpa*). The phenolic compounds were quantified using high performance liquid chromatography coupled to a diode-array detector HPLC-DAD and an ultra-performance liquid chromatograph (UPLC) with a Waters Acquity photodiode array detector (PDA) coupled to a Synapt G2 quadrupole time-of-flight (QTOF) mass spectrometer. Cyanidin-3-O-β-sambubioside (Cy-3-Sa) and cyanidin-3-O-glucoside (Cy-3-G) were the dominant anthocyanins in the fresh and freeze-dried Natal plum powder. Freeze drying did not affect the concentrations of both cyanidin compounds compared to the fresh fruit. Both cyanidin compounds, ellagic acid, catechin, epicatechin syringic acid, caffeic acid, luteolin, and quercetin O-glycoside from the ingested freeze-dried Natal plum powder was quite stable in the gastric phase compared to the small intestinal phase. Cyanidin-3-O-β-sambubioside from the ingested Natal plum powder showed bioaccessibility of 32.2% compared to cyanidin-3-O-glucoside (16.3%). The degradation of anthocyanins increased the bioaccessibility of gallic acid, protocatechuic acid, coumaric acid, and ferulic acid significantly, in the small intestinal digesta. The ferric reducing antioxidant power (FRAP), 2,2′-azino-bis-3-ethylbenzthiazoline-6-sulphonic acid (ABTS) activities, and inhibitory effect of α-glucosidase activity decreased in the small intestinal phase. Indigenous fruits or freeze-dried powders with Cy-3-Sa can be a better source of anthocyanin than Cy-3-G due to higher bioaccessibility in the small intestinal phase.

## 1. Introduction

Market knowledge and interest in nutritional quality, particularly in healthier foods, is currently apparent in many communities. Food suppliers are striving to develop innovative food products with functional powders. The projection is that global functional foods’ market size would increase in 2025 to US $275.77 billion, predominantly due to the increasing consumer demand for nutritional and fortifying food additives [1]. Furthermore, the market growth of global functional powdered drink concentrates increased by 7% during the period 2017–2021 [2]. Fruits with bright red, or pink to dark blue colours predominantly contain anthocyanins belonging to the phenolic group and show numerous health benefits due to their potent antioxidant properties [3].

The Natal plum (*Carissa macrocarpa*), an indigenous berry to South Africa, has an attractive red colour and is used in the preparation of jams, sauces, desserts, yoghurt, jellies, ice cream, and fruit leathers. It is also commonly grown in southern Florida, California, and the Caribbean islands. It is rich in anthocyanin derivatives cyanidin-3-O-β-sambubioside (Cy-3-Sa) and cyanidin-3-O-glucoside (Cy-3-G). The Natal plum also contains naringenin 4′-O-glucoside, quercetin 3-O-rhamnosyl-galactoside, quercetin 3-O-rhamnosyl-glucoside, and ascorbic acid. All of these compounds have health-promoting antioxidant activity [4]. Due to the highly perishable nature of the fruit and limited postharvest storage facilities, drying fruits after harvest enables long-term storage and provides a convenient mode of transportation and handling. Thermal drying negatively affects the attractive colour due to the polymerisation of monomeric anthocyanins [5]. However, freeze drying at low temperatures and vacuuming influence the structural properties (density, porosity, and texture), produce high-quality food powders, prevent the degradation of thermally sensitive nutritional compounds, and preserve the colour [6]. The functional and therapeutic effect depends on how much of the phenolics are bioavailable and bioaccessible in the intestinal phase [7], which is subject to the fruit matrix and location of phenolics in the fruit cells [8]. The fraction of the portion of the ingested compound that is released from its matrix in the small intestine, and that is available for effective absorption by the small intestine, can be defined as the bioaccessible ingested compound [9].

*In vitro* and *in vivo* models are used for assessing the bioavailability of functional compounds during digestion [10]. However, screening of the bioavailability of bioactive compounds using *in vitro* gastrointestinal models is less expensive and does not need ethical clearance. The antioxidant activities of phenolic compounds after *in vitro* gastrointestinal digestion of fruits and vegetables have been shown by many researchers [11,12,13].

However, few studies have focused on the bioavailability of traditional fruit functional powders using *in vitro* gastrointestinal models. *In vitro* digestion studies on orange [14] and pomegranate [11] juices showed lower bioavailability of certain phenolic compounds, which could pose limitations on consumers’ health. Although the use of functional powders as active ingredients for the development of functional food is very popular, there is no data available on the effect of *in vitro* gastrointestinal digestion on the bioavailability of polyphenols from freeze-dried Natal plum powder. Furthermore, freeze drying of apples caused cellular damage and changes in the food matrix, and influenced the release, bioaccessibility, and bioavailability of nutrients from the food matrix [15].

Therefore, considering the above mentioned, the objective of this study was to determine the impact of freeze drying on the different phenolic components, antioxidant properties, and their bioaccessibility during simulated gastrointestinal digestion.

## 2. Materials and Methods

### 2.1. Reagents and Standards

All the chemical reagents, standards, and solvents were purchased from Sigma Aldrich (Johannesburg, South Africa). All chemical reagents used were analytical grade.

### 2.2. Preparation of Natal Plum Fruit Powder (NPFP)

Natal plum fruits were harvested at edible maturity (from red to the dark red stage) at the Tshwane University of Technology, Pretoria campus (25°43′55.6″ S, 28°09′52.3″ E) gardens, Pretoria, South Africa, during summer (December 2019 and January 2020). The deseeded fruits were frozen and stored at −80 °C, while another portion of fruits was freeze-dried after 48 h of freezing using a Benchtop Freeze Dryer (VirTis Sp Scientific, Model # 2kBTES-55, Gardiner, NY, USA) at −47 °C to −53 °C for 7 days. The freeze dryer was maintained at pressures below 200 millitorr; the powder was finely ground using a mortar and pestle.

The moisture content of the fresh fruits was determined as the weight loss due to drying, while the moisture content of the resulting powder was determined by drying in an oven at 105 °C for 3 h [16]. The colour of the Natal plum fresh fruit and the powder was determined using a Minolta CR-400 chromameter (Minolta, Osaka, Japan), calibrated using a white tile. The L* value represented brightness where L* = 0 was black and L* = 100 white. The intensity of the red colour was represented by positive a* values, whereas the intensity of the yellow colour was represented by the positive b* value. For pH, 1.8 g of NPFP was rehydrated using 10 mL of distilled water (to mimic fresh fruit moisture content) for 90 min at 30 °C. The pH of the fresh and dried Natal plum was assessed using a digital pH meter. Data were the mean of three measurements.

### 2.3. In Vitro Digestion of NPFP

*In vitro* digestion was simulated according to the method previously described by Jara-Palacios [17]. The digestion model included three consecutive phases: oral, gastric, and small intestinal digestion. A simulated salivary fluid (SSF) pH 7 was prepared by mixing 15 mL of 0.5 M KCl, 3.7 mL of 0.5 M KH_2_PO_4_, 6.8 mL of 1 M NaHCO_3_, 0.5 mL of 0.15 M MgCl_2_(H_2_O)_6_, 0.06 mL of 0.5 M (NH_4_)_2_CO_3_, 0.09 mL of 6 M HCl, and 0.025 mL of 0.3 M CaCl_2_(H_2_O) then diluting with distilled water to make 400 mL of solution. A simulated gastric fluid (SGF) was prepared by mixing 6.9 mL of 0.5 M KCl, 0.9 mL of 0.5 M KH_2_PO_4_, 12.5 mL of 1 M NaHCO_3_, 11.8 mL of 2 M NaCl, 0.4 mL of 0.15 M MgCl_2_(H_2_O)_6_, 0.5 mL of 0.5 M (NH_4_)_2_CO_3_, 1.3 mL of 6 M HCl, and 0.005 mL of 0.3 M CaCl_2_(H_2_O) then diluting with distilled water to a total of 400 mL. The simulated intestinal fluid was prepared by mixing 6.8 mL of 0.5 M KCl, 0.8 mL of 0.5 M KH_2_PO_4_, 42.5 mL of 1 M NaHCO_3_, 9.6 mL of 2 M NaCl, 1.1 mL of 0.15 M MgCl_2_(H_2_O)_6_, 0.7 mL of 6 M HCl, and 0.04 mL of 0.3 M CaCl_2_(H_2_O) then diluting to make a 400 mL solution using distilled water.

Oral phase: A total of 5 g of the NPFP was weighed into a volumetric flask and 10 mL of SSF containing 75 U mL^−1^ amylase was added. The mixture was agitated at 170 rpm for 2 min at 37 °C.

Simulated gastric digestion: A 20 mL SGF was added to the bolus and the pH was adjusted to 2.5. The pepsin solution (2000 U mL^−1^ in 0.1 M HCl, pH 2.2) was added to initiate simulated gastric digestion. The mixture was incubated under agitation at 170 rpm for 2 h at 37 °C to mimic the gastric phase; a 10 mL sample was collected and cooled on ice for 10 min to stop reactions and stored at −80 °C for further observation.

Simulated small intestinal digestion: To the remaining mixture, 20 mL of SIF was added, and the pH adjusted to 7.5. Thereafter, 1.75 mL of a pancreatin solution (800 U mL^−1^), 20 mg of bovine bile extract, 20 mg of porcine bile extract, and 14 μL of 0.3 M CaCl_2_ were added, and the mixture was maintained under agitation at 37 °C for 2 h. The digestion ended by cooling the samples in an ice bath and immediately storing the sample at −80 °C for further analysis. A digestion blank containing enzymes but without the sample was also kept. The recovery and bioaccessibility were determined using Equations (1) and (2), respectively, as shown:

(1) $$\%\;\mathrm{Recovery}=(\frac{B_{GC}\;}{B_{ND}})\times 100$$

The *B_GC_* (mg kg^−1^)-was the phenolic compounds content in the gastric digesta and *B_ND_* (mg kg^−1^) -was the phenolic compounds content in the un-digested sample.

(2) $$\mathrm{Bioaccessibility}\;\%=(\frac{B_{SI}\;}{B_{ND}})\times 100$$

The *B_SI_* (mg kg^−1^) -was the phenolic compounds content in the intestinal digesta and *B_ND_* (mg kg^−1^) was the phenolic compounds content in the un-digested sample.

### 2.4. Extraction of Phenolic Compounds

Freeze-dried Natal plum extracts were produced using a previously described method, with slight modifications [4]. Each sample (100 mg) was extracted using 80:20 methanol/water (10 mL) and was ultrasonicated for 30 min at 30 °C then centrifuged (Hermle Z326k, Hermle Labortechnik GmbH, Wehingen, Germany) at 1000 g for 20 min at 4 °C. Extractions were performed in triplicate, and the extracts were used for the analysis of the total phenolic content (TPC), antioxidant capacity, antidiabetic activity, phenolic profile using HPLC-DAD, and UPLC/QTOF/MS.

### 2.5. Total Phenolic Content

The concentration of total phenol in the extracts was determined according to Ndou et al. [4] using 0.5 mL of diluted extract (100 μg mL^−1^), 2.5 mL of Folin–Ciocalteu reagent (diluted 10 times with water), and 2 mL of Na_2_CO_3_ (75 g L^−1)^. Quantitative measurements were performed and expressed as gallic acid equivalents (GAE) in mg kg^−1^.

### 2.6. Antioxidant Capacity

The antioxidant capacity was determined through methods previously described by Mpai et al. [18], using a 2,2-diphenyl-1-picrylhydrazyl (DPPH) radical scavenging assay. The reaction mixture contained a 250 μL DPPH (90 μM) solution and 28 μL of the sample in a 96-well microplate. Absorbance was read at 515 nm, with the results expressed as the concentration of antioxidants required to decrease the initial DPPH absorbance by 50% (IC_50_).

The production of the ABTS radical cation (ABTS ^+^) was by the reaction of the ABTS stock solution (7 mM) with 4.9 mM potassium persulphate at the ratio of 1:1 and leaving the mixture to stand in the dark at 25 °C for 12–16 h before use. Forty microliters of the sample (various concentrations) were added to 200 µL of the ABTS ^+^. The mixture, protected from the light, was incubated in a 96-well microplate reader at 37 °C for 10 min; the decrease in absorbance at 734 nm was measured and expressed as the IC_50_.

The antioxidant capacity ferric reducing antioxidant potential (FRAP) assay was performed according to Mpai et al. [18], using a freshly prepared FRAP solution in 25 mL of 0.3 M acetate buffer (pH 3.6), and 2.5 mL of 10 mM TPTZ [2,4,6-tris(2-pyridyl)-1,3,5-triazine] solution in 40 mM HCL, and 2.5 mL of 20 mM ferric chloride (FeCl_3_.6H_2_O) and FRAP solution (950 μL); the antioxidant capacity (FRAP) was expressed as mM of the vitamin C equivalent antioxidant capacity (VCEAC) per g.

### 2.7. In Vitro α-Glucosidase Inhibitory Activity

The *in vitro* α-glucosidase inhibitory activity of the hydroxymethanolic extracts was determined following a method outlined by Apostolidis and Lee [19], with some modifications. A mixture of 50 µL of the sample and 100 µL of 0.1 M phosphate buffer (pH 6.9) containing the glucosidase solution (1 U mL^−1^) was incubated in 96-well plates at 25 °C for 10 min. After pre-incubation, 50 µL of 5 mM, p-nitrophenyl-α-d-glucopyranoside solution in 0.1 M phosphate buffer (pH 6.9) was added to each well at timed intervals. The reaction mixtures were incubated at 25 °C for 5 min. Before and after incubation, a microplate reader recorded the absorbance at 405 nm (SpectraMax M5, Molecular Devices, San Jose CA, USA). The calculation of the glucosidase inhibitory activity, expressed as inhibition percent, was as follows:

(3) $$\mathrm{Inhibition}\;\%=(1-\frac{Abs\;sample}{Abs\;control})\times 100$$

where *Abs sample* and *Abs control* were defined as absorbance of the sample and absorbance of the control, respectively.

### 2.8. Quantification of Targeted and Untargeted Phenolic Metabolites

The targeted phenolic compounds (flavonoids and phenolic acids) extracted using the hydro-methanol extracts mentioned in 2.4 were quantified using HPLC-DAD, Model Flexar TM 89173-556 (PerkinElmer, Waltham, MA, USA), as described previously by Mpai et al. [18] and the supernatants were separated and filtered using PTFE membrane filters prior to phenolic profiling. Separation was achieved on a Waters HSS T3 C18, 2.1 × 100 mm, 1.7 μm column. The mobile phase A consisted of 0.1% formic acid, and mobile phase B included 0.1% formic acid in acetonitrile. The gradient started at 100% solvent A for 1 min and changed to 28% B for 22 min in a linear way, and thereafter to 40% B for 50 s and a wash step of 1.5 min at 100% B, followed by a re-equilibration to the initial conditions for 4 min. The flow rate was 0.3 mL/min, and the column temperature was maintained at 55 °C, with an injection volume of 3 μL. The reading of the chromatogram was at 320 nm (phenolic acids and flavonoids). The phenolic acids and flavonoids were identified and quantified using pure external standards. Supplementary Table S1 presents the regression equations Limit of detection (LOD) and Limit of quantification (LOQ) for compounds quantified using HPLC-DAD. A Waters ultra-performance liquid chromatograph (UPLC) with a Waters Acquity photodiode array detector (PDA) coupled to a Synapt G2 quadrupole time-of-flight (QTOF) mass spectrometer were used to detect and quantify the untargeted phenolic compounds (anthocyanin metabolites, flavonoid glycosides, and amino acid derivatives) as previously described for Natal plum fruits by Ndou et al. [4]. The UPLC was equipped with an electrospray ion source operating in negative ESI mode with a cone voltage of 15 V, with a desolvation temperature of 275 °C and a desolvation gas flow at 650 L/h. The quantified compounds were expressed as mg kg^−1^.

### 2.9. Statistical Analysis

All the analyses in this study were in triplicate and repeated twice. An analysis of variance (ANOVA) and Tukey’s multiple-range test were used to determine the significant differences at *p*-values < 0.05. A nonlinear regression “dose-response inhibition” determined the IC_50_ in DPPH, and ABTS activities. The calculation of Pearson’s correlation coefficients was used to determine the strength of the linear relationships between total phenolic content, phenolic compounds, and antioxidant activities.

## 3. Results and Discussion

### 3.1. Changes in Physicochemical Properties of Natal Plum during Freeze Drying

The changes in the physicochemical properties of the Natal plum during freeze drying are shown in Table 1. The fresh Natal plum was mainly composed of water (77.2%). Freeze drying removed all ice water and some bound water and reduced the moisture content of the Natal plum to levels that storage life can be extended without any microbial activity [20]. Moisture content below a 43–46% dry weight basis reduced the microbial activity in grape pomace [21]. The pH of the fresh Natal plum was not significantly different (*p* > 0.05) to that of NPFP. The colour of the freeze-dried product is an important quality parameter that influences consumer acceptance. The lightness (L*) responsible for the lightness of the fresh Natal plum increased, while the (a*) colour coordinate responsible for the red colour decreased during freeze drying. Conversely, freeze drying in conditions of high pressure (42 Pa and 132 Pa), due to the shrinkage of fruit tissues, resulted in a decline in the L* value of strawberries [21]. Freeze drying increased the b* value in NPFP compared to the fresh Natal plum, and a similar increase was observed in strawberries, and may be due to the prolonged freeze-drying time which might have allowed enzymatic browning to occur [21].

### 3.2. Characterisation, Identification of Phenolic Components from Natal Plum and NPFP

The fresh Natal plum and NPFP from HPLC-DAD contained three hydroxybenzoic acids (ellagic acid, gallic acid, protocatechuic acid), five hydroxycinnamic acids (p-coumaric acid, ferulic acid, caffeic acid, chlorogenic acid, syringic acid), four flavonols (catechin, epicatechin, kaempferol, quercetin), one flavanone (naringenin), two flavones (apigenin, luteolin), and the UPLC/QTOF/MS data showed three flavonol glycosides (quercitrin, quercetin 3-galactoside 7-rhamoside, and rutin). Supplementary Table S1 shows the identification and quantification of phenolic acids and flavonoids using HPLC-DAD. Supplementary Table S2 illustrates the phenolic metabolite profile of the 80:20 methanol: water extracts of nondigested, intestinal, and gastric digested NPFP generated using UPLC/QTOF/MS. The most abundant class of compounds identified were phenolic glycosides (66.67%), made up of anthocyanins and quercetin glycosides; also tentatively identified were amino acids (11.11%). The MS^2^ spectrum of peak 3 exhibited a base peak ion at *m*/*z* 285[M-H-162]^−^ which was a result of cleavage of the rhamnose unit from the parent ion. A secondary fragment observed at *m*/*z* 299 corresponded to the quercetin fragment. Peak 3 was tentatively identified as quercetin-3-rhamnose (quercitrin).

The MS spectrum of peak 4 showed a secondary fragment observed at *m*/*z* 125.0371, which corresponded to phyloglucinaldehyde and was a degradation product of cyanidin-based anthocyanins produced after the subsequent deglycosylation of the anthocyanin followed by the cleavage of the aglycone [22]. The fragment ions identified at *m*/*z* 285 [M-2H]^−^ and *m*/*z* 447 [M-2H]^−^ were consistent with the fragments cyanidin aglycone and cyanidin glycoside, respectively, of the negative mode [23]. Further, fragments consistent with the xylose unit (at *m*/*z* 149[M-H-285-147]^−^) and the glycosyl moiety (at *m*/*z* 147[M-H-285-149]^–^) were observed, and these were produced due to the subsequent loss of the cyanidin aglycone moiety from the parent ion, and the fragmentation of the sambubioside to its monosaccharide units. Ezzat et al. [24] also identified the fragment at *m*/*z* 285, which was consistent with cyanidin and was able to identify the compound as cyanidin-3-sambubioside. Thus, peak 4 was tentatively identified as cyanidin-3-sambubioside (Cy-3-Sa) (Supplementary Figures S1 and S2A). In the MS spectrum, peak 6 showed a molecular ion at *m*/*z* 449.1094 that produced MS^2^ fragmentation, and had secondary fragment ions at *m*/*z* 285 [M-2H]^−^ which was for the cyanidin aglycone produced after the cleavage of the glycose unit from the parent ion and another fragment ion at *m*/*z* 447[M-2H]^−^ (Supplementary Figure S1) consistent with cyanidin glycoside [23]. McDougall et al. [25] used the positive mode and observed the same fragment ion at *m*/*z* of 287[M]^+^ for the aglycone cyanidin in the anthocyanin cyanidin-3-O-glucoside. As such, peak 6 was identified tentatively as cyanidin-3-O-glucoside (Cy-3-G) (Supplementary Figures S1 and S2B). The ESI MS spectrum of peak 7 exhibited a molecular ion at *m*/*z* 609.1399. In its MSE spectrum, peak 7 had a base peak ion at *m*/*z* 300 [M-H-147-162] as a result of the successive loss of rhamnose and the galactose moiety. The fragment ion at *m*/*z* 300 indicated that the compound is a quercetin derivative [26]. Peak 7 was tentatively identified as quercetin-3-galactoside 7-rhamnoside. In the first order mass spectrum of peak 8, a molecular ion was observed at *m*/*z* 609.1445. The tandem mass spectrometry (MS/MS) spectrum of peak 8 had a base peak ion at *m*/*z* 300.0302[M-H-308]^–^ due to the loss of the rhamnose and glucosyl units from the parent ion. The fragment ion at *m*/*z* 300/301 is characteristic of quercetin derivatives. A secondary peak was observed at *m*/*z* 463 [M-H-146] as a result of the loss of the rhamnose moiety from the parent ion. Another secondary fragment observed at *m*/*z* 179 was due to retrocyclisation following fission of the C ring of quercetin [26]. As such, peak 7 was tentatively identified as quercetin-3-glucoside 7-rhamnoside (rutin). The MS spectrum of peak 9 exhibited peaks at *m*/*z* 463.0977. Similar to peak 7, the MSE spectrum of peak 8 had a base peak ion at *m*/*z* 300[M-H-146] due to the cleavage of a galactosyl residue from the parent ion. Observation of the peak at *m*/*z* 300 revealed that peak 8 was a quercetin derivative [26]. A secondary fragment observed at *m*/*z* 153 was due to the cleavage of the quercetin moiety. The identity of peak 8 was tentatively identified as quercetin 3 galactoside (hyperoside).

### 3.3. Changes in Total Phenolic Content, Phenolic Components, and Antioxidant Properties during Freeze Drying

Freeze drying caused a 12.04% loss of the total phenol content compared to the fresh fruit (Table 2). Similarly, freeze drying caused a loss of the total phenol content in starfruit, mango, papaya, and watermelon compared to their fresh samples [27]. The observed reduction in total phenolic compounds during the freeze-drying process was possibly due to the disruption of cells and decompartmentalization that had facilitated the contact and activity of enzymes [27].

Freeze drying reduced the Cy-3-Sa and Cy-3-G contents by 3.36 and 8.99%, respectively, compared to the fresh samples. However, the anthocyanin content of açaí (*Euterpe oleracea*), the Brazilian native fruit, increased during freeze drying compared to the fresh fruit [28]. It is probable that the structural changes caused by the ice formation and sublimation leading to cellular disruption facilitated the extraction of anthocyanins compounds [28]. Moreover, a strong degradation of Cy-3-G was observed during the freeze drying of blackcurrant pomace [29]. The total porosity, average pore radius, and water binding capacity of the freeze-dried Natal plum (FDNP) could have affected the solubility of solutes [30]. Freeze drying increased the concentrations of hydroxybenzoic acids (ellagic acid, gallic acid, protocatechuic acid), hydroxycinnamic acids (ferulic acid, caffeic acid, and syringic acid), and flavonoids (apigenin, quercetin, naringenin, and luteolin) (Table 2), which could be possibly due to the liberation of phenolic compounds from the matrix. However, flavonoid glycosides and flavonoids (epicatechin, kaempferol), and p-coumaric acid were reduced during the freeze-drying process. The long freeze-drying period could have also contributed to the loss of phenolic compounds; a previous study reported the stability of phenolic compounds when samples were freeze-dried for a shorter period [31].

Freeze drying reduced the FRAP activity significantly, while ABTS and DPPH scavenging activities remained unchanged compared to the fresh fruit (Table 2). This indicates that although there are differences in the total quantities of the different phenolic components released, the altered cellular structure could have released some compounds with higher release. Similarly, freeze drying decreased the FRAP activity in starfruits and mangos as compared to that of fresh samples [27]. In addition, FRAP activity strongly and positively correlated with coumaric acid, kaempferol, Cy-3-Sa, Cy-3-G, quercitrin (quercetin O-glycoside), catechin, and quercetin 3-O-rutinoside (Supplementary Table S3), and the loss of these compounds during freeze drying negatively affected the FRAP activity.

Moreover, a strong positive correlation existed between DPPH radical scavenging activity to caffeic acid, catechin, epicatechin, quercitin, luteolin Cy-3-Sa Cy-3-G, quercitrin, and quercetin 3-galactoside (Supplementary Table S3). ABTS radical scavenging activity also established a strong correlation with gallic acid, protocatechuic acid, p-coumaric acid, catechin, epicatechin Cy-3-Sa, Cy-3-G, and quercetin 3-galactoside (Supplementary Table S3). The results confirm that the higher concentration of anthocyanins and the variety of phenolics contributes to the antioxidant capacity of NPFP. Similarly, higher concentrations of anthocyanins and different phenolics in the crowberry showed a more potent antioxidant capacity compared to other berries [32].

### 3.4. Changes in Total Phenolic Content of NPFP during In Vitro Gastrointestinal Digestion

Table 2 illustrates the total phenols in nondigested NPFP and digested gastric and intestinal fractions of the sample. The total phenolic content of NPFP (nondigested) showed the highest amount of total phenolic content (2265.77 mg kg^−1^), the gastric fractions showed 1786.13 mg kg^−1^ and the lowest content was in the intestinal fraction (1399.18 mg kg^−1^). However, the percentage recovery of the total phenols in the gastric fraction was 71.5% with respect to the nondigested sample, and the bioaccessible amount in the intestinal fractions was 61.8%. A similar decrease in the total phenolic content in the gastric and intestinal phases compared to the nondigested sample was reported in crisphead lettuce [33]. In contrast, pomegranate juice extracted in 50% ethanol showed higher total phenol in the gastric fraction than the nondigested sample and the levels were more or less similar in both gastric and intestinal fractions [34]. The release of different phenolic compounds during the gastric and intestinal phases from the sample depends on the pH and types of other bioactive compounds in the plant material [33]. The decreasing trend in total phenols in the intestinal fraction compared to the gastric phase was also reported in strawberries [35], blackberries, blueberries, and raspberries [36], and persimmon juice [37], probably due to interactions with macro nutrients (proteins, carbohydrates), minerals, and fibre [38], or due to the production of other phenolic derivatives as a result of polymerisation and oxidation reactions [39]. Furthermore, the concentration of total phenols in the intestinal fraction of NPFP (1399.18 mg 100 g^−1^) was more or less similar to the amount reported in blackberries [40].

### 3.5. Release of Different Phenolic Components from NPFP during In Vitro Gastrointestinal Digestion

Anthocyanins were the most abundant polyphenols in NPFP. Cy-3-Sa was the predominant anthocyanin in nondigested NPFP, which contained Cy-3-Sa and Cy-3-G at 1631.4 mg kg^−1^ and 955.2 mg kg^−1^, respectively (Table 2). However, in the previous investigations Cy-3-G was higher in the fresh Natal plum than Cy-3-Sa; Cy-3-Sa increased with ripening at a higher rate than cyanidin glucoside. At the red stage in one season the contents were almost similar; therefore, seasonal variations and the ripeness stage affect the content [4], and one of the predominant anthocyanin compounds in strawberries [39]. Cy-3-Sa was also detected in maqui berry (*Aristotelia chilensis*) [41]. Table 2 also illustrates the composition of phenolic metabolites in the simulated gastrointestinal digestion model. Both Cy-3-Sa and Cy-3-G reduced to 1193.2 and 347.6 mg kg^−1^ in the gastric fraction, respectively. Cy-3-Sa showed a 73.14% recovery in the gastric fraction and the bioaccessible amount was 32.2% in the intestinal phase; whereas, Cy-3-G was recovered in the gastric fraction by 36.39%, with a 16.3% bioaccessible amount in the intestinal phase. A similar decrease in cyanidin derivatives, such as cyanidin-3-sophoricoside, cyanidin 3-(2^G^ glucosylrutinoside), and cyanidin-3-rutinoside were reported in the intestinal fraction of the raspberry extract [42].

Talavéra et al. [43] reported higher absorption of anthocyanin mono glycosides (glucosides) via the stomach, and the gastric fraction showed a 36.4% recovery of Cy-3-G in this study. Crozier et al. [44] stated that the anthocyanins hydrolyse in the intestine, and then they are absorbed in the gut. The stability of anthocyanin molecules and their molecular structure was affected by the pH of the gastrointestinal phase and the increase in the number of hydroxyl groups [45]. In general, anthocyanins are more stable in the acidic pH (2.2) in the stomach than the neutral conditions of the small intestine. The colour form of flavylium cation of the anthocyanin will be stable at low pH (2.2), while with pH 7.5, anthocyanins transform into the colourless chalcone pseudobase [46], and the C ring and the glycosidic bond in the anthocyanin molecule cleave and degrade to phenolic compounds with lower molecular weights [46]. There was no detection of chalcone pseudobase, and it probably would have decomposed into protocatechuic acid [46]. All of these possible events could have reduced the bioaccessibility of Cy-3-Sa (32.2%) and Cy-3-G (16.3%) at the intestinal phase. Oliveira et al. [46], in their investigation, showed a 2.5% recovery rate of Cy-3-G in strawberries, which was lower than the value (16.3%) obtained for NPFP. Furthermore, chalcone pseudobase could have decomposed into protocatechuic acid or ferulic acid and coumaric acid from its B ring in the intestinal fraction [46]. The degradation of anthocyanins was concurrent with an increase in protocatechuic acid (88.6 mg kg^−1^), coumaric acid (85.7 mg kg^−1^), and ferulic acid (73.5 mg kg^−1^), in the intestinal fraction, which resulted in an increase in their bioaccessibility in this study. The application of encapsulation techniques could protect the anthocyanins from degradation in the small intestine [47].

Ellagic acid concentration increased to 49.6 mg kg^−1^ in the gastric fraction compared to the nondigested NPFP and there was lower concentration obtained in the intestinal fraction (11.3 mg kg^−1^). Percentage recovery of ellagic acid in the gastric fraction and its bioacccesibility was 116.9% and 26.7%, respectively, compared to the nondigested NPFP. Increased recovery of ellagic acid in the gastric fraction was probably due to the further hydrolysis of ester bonds and release of ellagitannins due to the lower pH environment during simulated digestion [48]. Caffeic and syringic acids reduced in the gastric and intestinal fractions significantly. The bioaccessibility of phenolics during digestion depends on their stability when exposed to the different pH, structure of the compound, fruit matrix, solubility, and some degradation or glycosylation and esterification with other compounds [49]. An increase in gallic acid after intestinal digestion may be due to the presence of gallotannins, which spontaneously degrade at alkaline pH [50].

The gastric fraction of NPFP showed significantly lower (*p* < 0.005) concentrations of flavonoid aglycones, such as kaempferol (70.9 mg kg^−1^), quercetin (88.6 mg kg^−1^), naringenin (22.3 mg kg^−1^), and apigenin (5.9 mg kg^−1^), compared to the nondigested NPFP, and their bioaccessibility increased in the intestinal fraction. However, Rha et al. [51] showed that the concentration of flavanol aglycon kaempferol from green tea did not change significantly in the gastric fraction, but significantly declined in the intestinal fraction in the presence of pancreatin. Rodríguez and Tironi [52] showed an increase in kaempferol concentration in the gastrointestinal fraction of amaranth (*Amaranthus* manteggazianus) flour. The type of solvents (e.g., methanol/water or methanol/HCl) used during the extraction after gastrointestinal digestion, greatly influenced the concentration and the recovery of flavonoid aglycones [52]. In addition, as previously reported by Bouayed et al. [12] the increase in phenolic compounds in the intestinal fraction could be due to the release of phenolics bound to the matrix by the action of the intestinal digestive enzyme (pancreatin). Similar increases in flavonoids were reported in the frozen pulp of umbu-cajá (*Spondias tuberosa* x *Spondias mombin*) after gastrointestinal digestion [53]. The pH changes between the gastric and intestinal phases did not affect the luteolin. It may be necessary to investigate the structure of Natal plum cells and location phenolics to explain this phenomenon properly. The stability of catechin and epicatechin is pH-dependent and they are stable in the acidic solution [54], which could be the reason for the higher catechins concentration in the gastric phase.

The acidic pH affected the quercetin glycosides in the gastric fraction in this study, and their bioaccessible amounts in the intestinal fraction also varied based on the type of quercetin glycoside. Sugar moieties attached to the flavonoids during glycosylation prevent their degradation by providing a protective effect during digestion [55]. The sugar flavonol bond (β-glycosidic bond) was resistant to the hydrolysis by the pancreatic enzyme. The bioavailability of the ingested quercetin also depends on its dietary source, and the bioavailability of quercetin glucosides from onion powder was much higher than the other types of quercetin glycosides from apples, including quercetin rutinoside [56]. Therefore, the amount of quercetin present in the intestinal fraction depends mainly on the type and position of sugar moiety. Furthermore, due to exposure to acidic conditions, the rutin molecule (Quercetin 3-rutinoside) could have cleaved from the linkage with the sugar and generated the quercetin [57], which could have been the reason for the observed increase in flavonoid aglycon quercetin in the intestinal digesta in this study.

### 3.6. Principal Component Analysis

**Figure 1:**
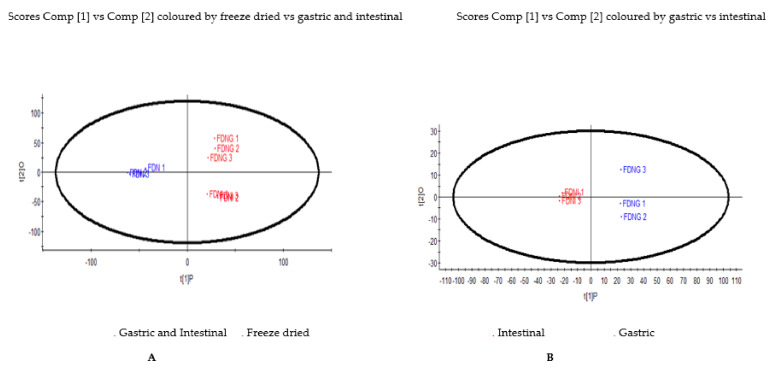
(**A**): Score plot of principal component analysis (unsupervised) based on UPLC–Q-TOF/MS spectra of the Natal plum freeze-dried fruit powder during simulated gastrointestinal digestion. Two cluster groups showing FDN on the left-hand side, and FDNG and FDNI on the right-hand side. (**B**): Score plot of principal component analysis (unsupervised) of the Natal plum freeze-dried fruit powder in the gastric and intestinal fraction. FDN: Freeze-dried Natal plum, FDNG: Freeze-dried Natal plum in the gastric fraction; FDNI: Freeze-dried Natal plum in the intestinal fraction.

**Figure 2:**
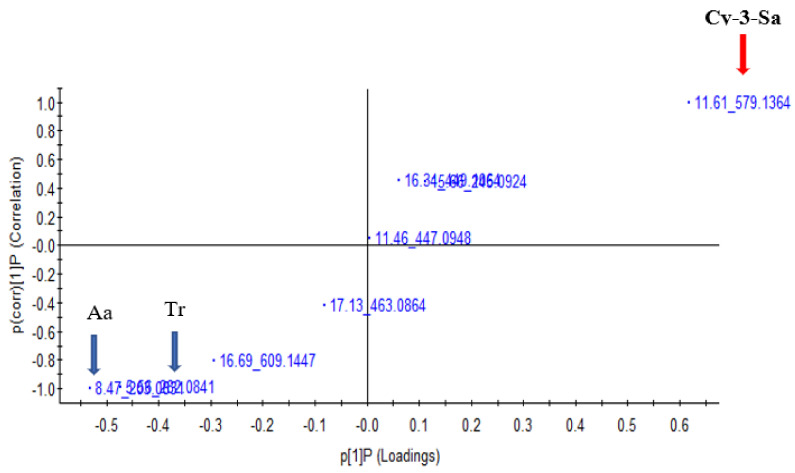
Score plot of orthogonal partial least squares discriminant analysis of UPLC–Q-TOF/MS spectra of digested Natal plum freeze-dried powder in the gastric and intestinal fractions. Each sample set includes three replicates: Cyanidin sambubioside (*m*/*z* 579.13, retention time 11.61); Aa-amino acid derivative; (*m*/*z*) 203.08, retention time 8.47); and tryptophan (*m*/*z*) 282.08, retention time 5.56).

The illustration of the differences in distribution of the non-targeted phenolic metabolic profiles of the NPFP that underwent simulated gastric and intestinal digestion was presented using an unsupervised principal component analysis (PCA) approach, from the data set obtained by the UPLC/QTOF/MS analysis. The PCA plot highlighted two district clusters based on the phenolic metabolites shown in Figure 1A; NPFP were separated from the samples of the gastric and intestinal fractions, and in Figure 1B, the samples from gastric fractions were separated from the intestinal fraction. The performing of an orthogonal projections to latent structures discriminant analysis (OPLS-DA) for metabolomics data was to exhibit a quantitative relationship between the different simulated gastrointestinal fractions (treatments) and the metabolites, to identify the biomarker candidates responsible for the separation between the gastrointestinal treatments. The values of R^2^ X and Q^2^ of the cross-validation in the OPLS-DA score plot for NPFP samples for gastro digestion fractions were 0.962 and 0.955, respectively, and provided reliable fitness. The upper right quadrant of the S-plot (Figure 2) illustrated the higher concentration of phenolic metabolite Cy-3-Sa ([(M-H) 579.13, RT 11.61) in the gastric fraction, while the lower left quadrant showed the higher concentration of amino acid derivative ([(M-H) 203.08, RT 8.47) and tryptophan ([(M-H) 282.08, RT 5.56) in the intestinal fraction, which may have been released as proteins were enzymatically hydrolysed.

**Figure 3:**
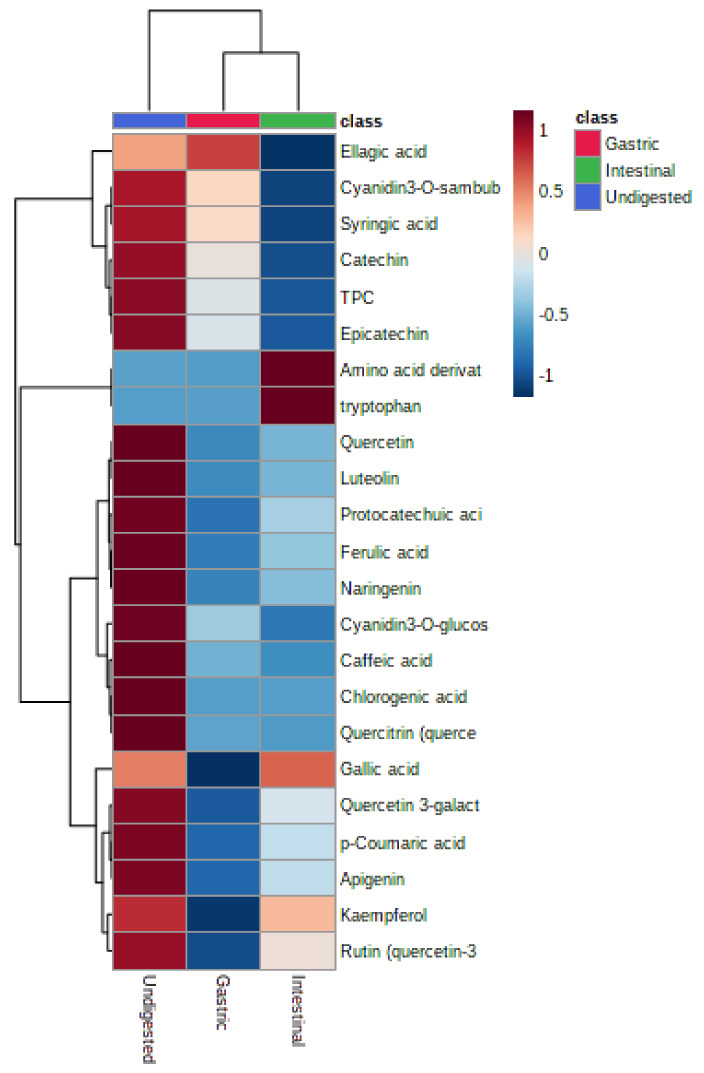
Heat map of 20 phenolic metabolites in the gastrointestinal fraction compared to the Natal plum freeze-dried powder organised in a hierarchical clustering.

Figure 3 represents the heat map for metabolites of NPFP nondigested and digested Natal plum powder via the *in vitro* digestion model. The pattern and magnitude of the colour intensity (hue) relates to the visualisation of response intensities of the compounds detected in the Natal plum. The data showed that all phenolic metabolites, except ellagic acid levels, decreased during gastric digestion. Conversely, amino acid derivatives, and tryptophan, were at higher levels in the intestinal fraction.

### 3.7. Antioxidant Capacity of Gastric and Intestinal Fraction of NPFP

DPPH scavenging activity and ABTS capacity decreased in the gastric fraction compared to the nondigested NPFP, while at the intestinal phase, the scavenging activity increased significantly (Table 2). However, the FRAP activity was similar in the gastric fraction and the nondigested sample but decreased in the intestinal fraction. Therefore, in this study there was a reduction in DPPH, ABTS, and FRAP activities in the intestinal fraction, compared to the original methanolic fraction of the NPFP, due to the lower concentration of phenolic compounds. Confirmation of this was by the positive correlation with FRAP activity (Supplementary Table S3). Reportedly, the pH changes between the gastric and intestinal phase also affected the antioxidant capacity [12]. Tagliazucchi et al. [50] showed grape extracts rich in phenolics (e.g., quercetin) providing more protection in the intestine than the stomach phase and showing a higher antioxidant capacity when measured in ABTS; there was no similar trend seen in this study. The antioxidant capacity of phenols is mainly due to their redox properties, which allows them to act as reducing agents, hydrogen donors, and singlet oxygen quenchers [58]. Furthermore, free radical scavenging and the antioxidant capacity of phenols mainly depend on the number and position of hydrogen-donating hydroxyl groups on the aromatic ring of the phenol molecules [59]. Moreover, the release of amino acids or sugars from original food components also could have increased the values of the total antioxidant capacity assays.

### 3.8. α-Glucosidase Inhibitory Activity of Digested and Nondigested Natal Plum

**Figure 4:**
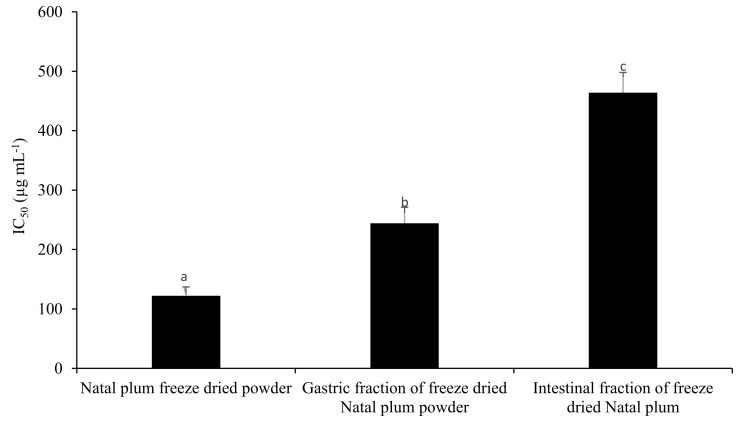
Effect of *in vitro* digestion or freeze drying on the inhibition of α-glucosidase activity. Bars with the same letter are not significantly different at *p* < 0.05.

α-glucosidase, a key enzyme for carbohydrate digestion, has been recognised as a therapeutic target for the modulation of postprandial hyperglycaemia, which is the earliest metabolic abnormality to occur in type 2 diabetes mellitus [19]. The inhibition capacity of NPFP was higher in the nondigested freeze-dried sample than the fractions at the gastric and intestinal phases (Figure 4). There was a strong positive correlation of the α-glucosidase IC_50_ with Cy-3-Sa, Cy-3-G, protocatechuic acid, caffeic acid, catechin, epicatechin, quercitin, naringenin, luteolin, quercitrin, and quercetin 3-O-rutinoside (Supplementary Table S3), indicating that when these phenolics increased, the inhibition also increased. Anthocyanin rich food consumption is beneficial in reducing the postprandial hyperglycaemia via inhibitory effects against α-amylase and α-glucosidase, which reduces the glucose transport across the small intestine [60]. Red cabbage of the Koda variety (IC_50_ value of 3.87 mg mL^−1^), of *Momordica charantia* var. *charantia* (IC_50_ value 0.298 mg mL^−1^) and *M. charantia* var. *muricata* (IC_50_ value 0.292 mg mL^−1^) showed lower inhibitory activity than the activity reported with NPFP [61]. Tadera et al. [62] reported the presence of an unsaturated C ring, hydroxylation on the C ring at the 3-position, and an increase in the number of hydroxyl groups on the B ring enhanced the inhibitory activity of α-glucosidase.

## 4. Conclusions

To the best of our knowledge, the findings on changes in the phenolic components and antioxidants of freeze-dried indigenous fruit powders are limited during different simulated *in vitro* digestion phases. The simulated gastrointestinal digestion affected the stability of the anthocyanin. Bioactivity of FRAP, DPPH, and ABTS, and the bioaccessibility percentage of Cy-3-Sa and Cy-3-G decreased in the intestinal fraction of *in vitro* simulated digestion compared to the nondigested sample. The inhibitory effect of α-glucosidase activity decreased greatly in the gastric and intestinal phases. Indigenous fruits or freeze-dried powders with Cy-3-Sa can be a better source of anthocyanin than Cy-3-G due to higher bioaccessibility in the small intestinal phase. Furthermore, it is necessary to find ways to improve the bioavailability and accessibility of Cy-3-Sa and Cy-3-G to benefit functional foods, such as Natal plums, with improved bioactivities. Thus, it is important to encourage the use of innovative techniques such as encapsulation for better delivery of these bioactive compounds in the human body to exploit their best abilities. Future research should focus on the *in vitro* digestion of freeze-dried Natal plum powder with other foodstuffs like indigenous vegetables and maize meal to see the proportion of anthocyanins that are bioaccessible compared to the freeze-dried powder.

## Figures and Tables

**Table 1:** Physicochemical characteristics of the fresh Natal plum and NPFP.

Physicochemical Property	Fresh Natal Plum	NPFP
Moisture content (g /100 g)	77.2 ± 1.3 ^a^	6.4 ± 0.8 ^b^
pH	3.19 ± 0.1 ^a^	3.21 ± 0.1 ^a^
Colour coordinates		
L*	36.6 ± 1.2 ^b^	41.4 ± 2.2 ^a^
a*	20.2 ± 2.1 ^a^	17.4 ± 0.7 ^b^
b*	6.3 ± 0.5 ^a^	7.7 ± 0.9 ^a^

Means ± standard deviation followed by the same letter within the row are not significantly different at *p* < 0.05.

**Table 2:** Changes in phenolic components in Natal plum fruits during freeze drying and gastrointestinal digestion on a dry weight basis.

	Fresh Natal Plum (DW)	Freeze-Dried Natal Plum (Non-Digested)	% Loss	Gastric Digested Natal Plum (DW)	Recovery %	Intestinal Digested Natal Plum (DW)	Bioaccessibility (%)
**TPC (mg GAE kg^−1^)**	2576.05 ^a^ ± 4.85	2265.77 ^a^ ± 1.23 *	12.04	1786.13 ^b^ ± 0.08	78.8	1399.18 ^c^ ± 0.16	61.8
**Phenolic components (mg kg^−^^1^)**							
*Anthocyanins*							
Cyanidin-3-O-sambubioside	1688.1 ^a^ ± 4.3	1631.4 ^a^ ± 1.2	3.36	1193.2 ^b^ ± 1.5	73.1	525.3 ^c^ ± 3.1	32.2
Cyanidin-3-O-glucoside	1049.5 ^a^± 2.2	955.2 ^a^ ± 1.9	8.99	347.6 ^b^ ± 1.5	36.4	155.7 ^c^ ± 1.8	16.3
*Hydroxybenzoic acids*							
Ellagic acid	26.0 ^c^ ± 0.4	42.4 ^b^ ± 0.1	+63.07	49.6 ^a^ ± 0.5	116.9	11.3 ^d^ ± 0.2	26.7
Gallic acid	3.8 ^c^ ± 0.4	5.5 ^b^ ± 0.1	+44.74	2.1 ^c^ ± 0.1	38.2	8.8 ^a^ ± 0.1	160
Protocatechuic acid	158.8 ^b^ ± 5.0	167.6 ^a^ ± 0.9	+5.54	59.6 ^d^ ± 0.2	35.6	88.6 ^c^ ± 0.5	52.9
*Hydroxycinnamic acids*							
p-Coumaric acid	226.4 ^a^ ± 5.2	140.3 ^b^ ± 1.6	38.03	55.4 ^d^ ± 0.5	39.5	85.7 ^c^ ± 0.2	61
Ferulic acid	126.4 ^b^ ± 4.6	165.1 ^a^ ± 0.3	+30.61	49.6 ^d^ ± 0.3	30	73.5 ^c^ ± 0.3	44.5
Caffeic acid	120.1 ^b^ ± 1.26	168.4 ^a^ ± 0.1	+40.22	53.8 ^c^ ± 0.5	31.9	40.8 ^d^ ± 0.4	24.2
Syringic acid	100.4 ^a^ ± 4.2	62.2 ^b^ ± 0.2	38.05	47.5 ^c^ ± 0.6	76.4	26.5 ^d^ ± 0.2	42.6
*Flavonoids*							
Catechin	497.7 ^a^ ± 9.7	491.8 ^a^ ± 2.3	0.0	354.1 ^b^ ± 2.8	72	210.4 ^c^ ± 1.4	42.8
Epicatechin	21.3 ^a^ ± 0.6	19.3 ^a^ ± 0.2	9.39	12.2 ^b^ ± 0.2	63	6.7 ^c^ ± 0.2	34.7
Kaempferol	493.5 ^a^ ± 2.3	333.5 ^b^ ± 1.2	32.42	70.9 ^d^ ± 3.5	21.3	259.9 ^c^ ± 3.1	21.3
Quercetin	210.2 ^b^ ± 0.8	279.7 ^a^ ± 1.4	+33.06	88.6 ^d^ ± 5.9	31.7	112.1 ^c^ ± 0.9	40
Naringenin	37.4 ^b^ ± 1.3	48.3 ^a^ ± 0.2	+29.14	17.2 ^c^ ± 0.1	35.6	22.3 ^c^ ± 0.2	46
Apigenin	4.69	9.7 ^a^ ± 0.6	+106.82	3.8 ^c^ ± 0.1	39	5.9 ^b^ ± 0.1	60.8
Luteolin	3.4 ^b^ ± 0.5	4.6 ^a^ ± 0.1	+26.09	1.3 ^c^ ± 0.1	28.3	1.7 ^c^ ± 0.1	36.9
*Flavonoid glycoside*							
Quercitrin (quercetin-O-glycoside)	30.5 ^a^ ± 1.2	5.3 ^b^ ± 1.9	82.62	1.0 ^c^ ± 0.1	18.9	0.9 ^c^ ± 0.1	16.9
Quercetin-3-galactoside 7-rhamnoside	73.6 ^a^ ± 1.6	7.8 ^b^ ± 0.6	89.40	0.9 ^d^ ± 0.1	11.5	3.8 ^c^ ± 0.1	48.7
Quercetin 3-O-rutinoside (Rutin)	50.4 ^a^ ± 0.98	34.6 ^b^ ± 1.8	31.35	0.9 ^d^ ± 0.1	25	18.5 ^c^ ± 1.4	53.5
Amino Acid components (mg kg^−1^)							
Amino acid derivatives	ND	0.2 ^b^ ± 0.1		0.08 ^c^ ± 0.4	40	7.39 ^a^ ± 0.05	3695
Tryptophan	ND	0.01 ^b^ ± 0.0		0.03 ^b^ ± 0.0		9.12 ^a^ ± 0.2	912
**Antioxidant capacity**							
DPPH (IC_50_ for mg mL^−1^)	0.77 ^c^ ± 0.09	0.81 ^c^ ± 0.03		0.94 ^a^ ± 0.18		1.15 ^a^ ± 0.08	
ABTS (IC_50_ mg mL^−1^)	0.65 ^b^ ± 0.03	0.69 ^b^ ± 0.12		0.717 ^b^ ± 0.05		0.835 ^a^ ± 0.16	
FRAP (mM VCEAC 100 g^−1^)	603.5 ^a^ ± 4.3	493.54 ^b^ 603.5 ^a^ ± 1.40		482.54 ^b^ ± 1.11		399.42 ^c^ ± 0.88	

Means followed by the same letter within the row are not significantly different at *p* < 0.05; * standard deviation; VCEAC: vitamin C equivalent antioxidant capacity.

## Data Availability

Data is contained within the article or supplementary material.

## Supplementary Materials

The following are available online at https://www.mdpi.com/article/10.3390/foods10061420/s1, Figure S1. showing the ESI negative mode BPI chromatogram of the full chromatogram of the hydromethanol extract of Natal plum fruits overlaid with an expansion of the region 10.30 min to 17.00 min of the chromatogram which shows the anthocyanins cyanidin-3-O-β-sambubioside (A) at *m/z* 579.1356 retention time 11.56 min and cyanidin-3-O-glucoside (B) at *m/z* 449.1069 retention time 16.22 min; Figure S2A. Cyanidin-3-O-glucoside structure and MS/MS fragmentation; Figure S2B. Cyanidin-3-O-β-sambubioside structure and MS/MS fragmentation pattern; Table S1 Phenolic identification and quantification using HPLC-DAD; Table S2. Tentative peak identification of the compounds detected in the hydromethanol extract of undigested, gastric and intestinal digested Natal plum freeze dried powder; Table S3. Pearson’s correlation coefficient of different phenolic components antioxidant and α-glucosidase activities.

## References

[1] Report Link 2021 Functional Foods Market Size, Share & Trends Analysis Report by Ingredient, by Product, by Application and Segment Forecasts, 2019–2025. https://www.researchandmarkets.com/reports/4764576/functional-foods-market-size-share-and-trends.

[2] Business Wire Technovo Market Report 2021 Global Functional Powder Drinks Concentrate Market: Growth Analysis and Forecast by Technavio. https://www.businesswire.com/news/home/20170313006089/en/Global-Functional-Powder-Drinks-Concentrates-Market-Growth-Analysis-and-Forecast-by-Technavio.

[3] Khoo H.E., Azlan A., Tang S.T., Lim S.M. (2017). Anthocyanidins and anthocyanins: Colored pigments as food, pharmaceutical ingredients, and the potential health benefits. Food Nutr. Res..

[4] Ndou A., Tinyani P.P., Slabbert R.M., Sultanbawa Y., Sivakumar D. (2019). An integrated approach for harvesting Natal plum (*Carissa macrocarpa*) for quality and functional compounds related to maturity stages. Food Chem..

[5] Kara Ş., Erçelebi E.A. (2013). Thermal degradation kinetics of anthocyanins and visual colour of Urmu mulberry (*Morus nigra* L.). J. Food Eng..

[6] Krokida M.K., Maroulis Z.B., Saravacos G.D. (2001). The effect of the method of drying on the colour of dehydrated products. Int. J. Food Sci. Technol..

[7] Melini V., Melini F., Acquistucci R. (2020). Phenolic compounds and bioaccessibility thereof in functional pasta. Antioxidants.

[8] Tomas M., Beekwilder J., Hall R.D., Simon C.D., Sagdic O., Capanoglu E. (2018). Effect of dietary fiber (inulin) addition on phenolics and *in vitro* bioaccessibility of tomato sauce. Food Res. Int..

[9] da Silva E.N., de Farias L.O., Cadore S. (2018). The total concentration and bioaccessible fraction of nutrients in purées, instant cereals and infant formulas by ICP OES: A study of Dietary Recommended Intakes and the importance of using a standardized *in vitro* digestion method. J. Food Compos. Anal..

[10] Promchan J., Shiowatana J.A. (2005). dynamic continuous-flow dialysis system with on-line electrothermal atomic-absorption spectrometric and pH measurements for *in vitro* determination of iron bioavailability by simulated gastrointestinal digestion. Anal. Bioanal. Chem..

[11] Pérez-Vicente A., Gil-Izquierdo A., García-Viguera C. (2002). *In vitro* gastrointestinal digestion study of pomegranate juice phenolic compounds, anthocyanins, and vitamin C. J. Agric. Food Chem..

[12] Bouayed J., Hoffmann L., Bohn T. (2011). Total phenolics, flavonoids, anthocyanins and antioxidant activity following simulated gastro-intestinal digestion and dialysis of apple varieties: Bioaccessibility and potential uptake. Food Chem..

[13] Rodríguez M., Rojas-Graü M.A., Elez-Martínez P., Martín-Belloso O. (2013). Changes in vitamin C, phenolic, and carotenoid profiles throughout *in vitro* gastrointestinal digestion of a blended fruit juice. J. Agric. Food Chem..

[14] Gil-Izquierdo A., Gil M.I., Ferreres F., Tomás-Barberán F.A. (2001). *In vitro* availability of flavonoids and other phenolics in orange juice. J. Agric. Food Chem..

[15] Dalmau M.E., Bornhorst G.M., Eim V., Rosselló C., Simal S. (2017). Effects of freezing, freeze drying and convective drying on *in vitro* gastric digestion of apples. Food Chem..

[16] Kuljarachanan T., Chiewchan N., Devahastin S. (2019). Mechanical Grinding Effects on Health-Related Functional Properties of Dietary Fiber Powder from White Cabbage By-products. J. Adv. Agric. Technol..

[17] Jara-Palacios M.J., Gonçalves S., Hernanz D., Heredia F.J., Romano A. (2018). Effects of *in vitro* gastrointestinal digestion on phenolic compounds and antioxidant activity of different white winemaking by products extracts. Food Res. Int..

[18] Mpai S., Du Preez R., Sultanbawa Y., Sivakumar D. (2018). Phytochemicals and nutritional composition in accessions of Kei-apple (*Dovyalis caffra*): Southern African indigenous fruit. Food Chem..

[19] Apostolidis E., Lee C.M. (2010). *In vitro* potential of *Ascophyllum nodosum* phenolic antioxidant-mediated α-glucosidase and α-amylase inhibition. J. Food Sci..

[20] Rezaei F., Vandergheynst J.S. (2010). Critical moisture content for microbial growth in dried food-processing residues. J. Sci. Food Agric..

[21] Hammami C., René F. (1997). Determination of freeze-drying process variables for strawberries. J. Food Eng..

[22] Sinela A., Rawat N., Mertz C., Achir N., Fulcrand H., Dornier M. (2017). Anthocyanins degradation during storage of *Hibiscus sabdariffa* extract and evolution of its degradation products. Food Chem..

[23] Sun J., Lin L., Chen P. (2012). Study of the mass spectrometric behaviors of anthocyanins in negative ionization mode and its applications for characterization of anthocyanins and non-anthocyanin polyphenols. Rapid Commun. Mass. Spectrom..

[24] Ezzat S.M., Salama M.M., Seif el-Din S.H., Saleh S., El-Lakkany N.M., Hammam O.A., Botros S.S. (2016). Metabolic profile and hepatoprotective activity of the anthocyanin-rich extract of *Hibiscus sabdariffa* calyces. Pharm Biol..

[25] McDougall G.J., Dobson P., Smith P., Blake A., Stewart D. (2005). Assessing potential bioavailability of raspberry anthocyanins using an *in vitro* digestion system. J. Agric. Food Chem..

[26] Mashitoa F.M., Shoko T., Shai J.L., Slabbert R.M., Sivakumar D. (2021). Changes in phenolic metabolites and biological activities of pumpkin leaves (*Cucurbita moschata* Duchesne ex Poir.) during blanching. Front. Nutr..

[27] Shofian N.M., Hamid A.A., Osman A., Saari N., Anwar F., Pak Dek M.S., Hairuddin M.R. (2011). Effect of freeze-drying on the antioxidant compounds and antioxidant activity of selected tropical fruits. Int. J. Mol. Sci..

[28] Lucas B.F., Zambiazi R.C., Costa J.A.V. (2018). Biocompounds and physical properties of açaí pulp dried by different methods. LWT Food Sci. Technol..

[29] Michalska-Ciechanowska A., Wojdyło A., Lech K., Łysiak G.P., Figiel A. (2017). Effect of different drying techniques on physical properties, total polyphenols and antioxidant capacity of blackcurrant pomace powder. LWT Food Sci. Technol..

[30] Leusink G.J., Kitts D.D., Yaghmaee P., Durance T. (2010). Retention of Antioxidant Capacity of Vacuum Microwave Dried Cranberry. J. Food Sci..

[31] De Torres C., Díaz-Maroto M.C., Hermosín-Gutiérrez I., Pérez-Coello M.S. (2010). Effect of freeze-drying and oven-drying on volatiles and phenolics composition of grape skin’. Anal. Chim. Acta..

[32] Bae H.S., Kim H.J., Kang J.H., Kudo R., Hosoya T., Kumazawa S., Ahn M. (2015). Anthocyanin profile and antioxidant activity of various berries cultivated in Korea. Nat. Prod. Commun..

[33] Ketnawa S., Suwannachot J., Ogawa Y. (2020). *In vitro* gastrointestinal digestion of Crisphead lettuce: Changes in bioactive compounds and antioxidant potential. Food Chem..

[34] Fawole O.A., Opara U.L. (2016). Stability of total phenolic concentration and antioxidant capacity of extracts from pomegranate co-products subjected to *in vitro* digestion. BMC Compl. Altern. Med..

[35] Ariza M.T., Forbes-Hernandez T.Y., Giampieri F., Gasparrini M., Soria C., Martínez-Ferri E., Battino M. (2017). Effects of *in vitro* gastrointestinal digestion on strawberry polyphenols stability. Acta. Hortic..

[36] Marhuenda J., Alemán M.D., Gironés-Vilaplana A., Pérez A., Caravaca G., Figueroa F., Zafrilla P. (2016). Phenolic composition, antioxidant activity, and *in vitro* availability of four different berries. J. Chem..

[37] Martínez-Las Heras R., Pinazo A., Heredia A., Andrés A. (2017). Evaluation studies of persimmon plant (*Diospyros kaki*) for physiological benefits and bioaccessibility of antioxidants by *in vitro* simulated gastrointestinal digestion. Food Chem..

[38] Alminger M., Aura A.M., Bohn T., Dufour C., El S.N., Gomes A., Santos C.N. (2014). *In vitro* models for studying secondary plant metabolite digestion and bioaccessibility. Compr. Rev. Food Sci. Food Saf..

[39] Gil-Izquierdo A., Zafrilla P., Tomás-Barberán F.A. (2002). An *in vitro* method to simulate phenolic compound release from the food matrix in the gastrointestinal tract. Eur. Food Res. Technol..

[40] da Silva F.L., Escribano-Bailón M.T., Alonso J.J.P., Rivas-Gonzalo J.C., Santos-Buelga C. (2007). Anthocyanin pigments in strawberry. LWT Food Sci. Technol..

[41] Schreckinger M.E., Lotton J., Lila M.A., de Mejia E.G. (2010). Berries from South America: A Comprehensive Review on Chemistry, Health Potential, and Commercialization. J. Med. Food.

[42] Hao Y., Yang J., Cui J., Fan Y., Li N., Wang C., Dong Y. (2021). Stability and mechanism of phenolic compounds from raspberry extract under *in vitro* gastrointestinal digestion. LWT Food Sci. Technol..

[43] Talavera S., Felgines C., Texier O., Besson C., Lamaison J.L., Rémésy C. (2003). Anthocyanins are efficiently absorbed from the stomach in anesthetized rats. J. Nutr..

[44] Crozier A., Jaganath I.B., Clifford M.N. (2009). Dietary phenolics: Chemistry, bioavailability and effects on health. Nat. Prod. Rep..

[45] Kamonpatana K., Failla M.L., Kumar P.S., Giusti M.M. (2014). Anthocyanin structure determines susceptibility to microbial degradation and bioavailability to the buccal mucosa. J. Agric. Food Chem..

[46] Chen Y., Chen H., Zhang W., Ding Y., Zhao T., Zhang M., Yang L. (2019). Bioaccessibility and biotransformation of anthocyanin monomers following *in vitro* simulated gastric-intestinal digestion and *in vivo* metabolism in rats. Food Funct..

[47] Oliveira G., Tylewicz U., Dalla Rosa M., Andlid T., Alminger M. (2019). Effects of pulsed electric field-assisted osmotic dehydration and edible coating on the recovery of anthocyanins from *in vitro* digested berries. Foods.

[48] Oidtmann J., Schantz M., Mäder K., Baum M., Berg S., Betz M., Richling E. (2012). Preparation and comparative release characteristics of three anthocyanin encapsulation systems. J. Agric. Food Chem..

[49] Mosele J.I., Macià A., Romero M.P., Motilva M.J. (2016). Stability and metabolism of Arbutus unedo bioactive compounds (phenolics and antioxidants) under *in vitro* digestion and colonic fermentation. Food Chem..

[50] Tagliazucchi D., Verzelloni E., Bertolini D., Conte A. (2010). *In vitro* bio-accessibility and antioxidant activity of grape polyphenols. Food Chem..

[51] Rha C.S., Seong H., Jung Y.S., Jang D., Kwak J.G., Kim D.O., Han N.S. (2019). Stability and fermentability of green tea flavonols in *in vitro*-simulated gastrointestinal digestion and human faecal fermentation. Int. J. Mol. Sci..

[52] Rodríguez M., TironI V.A. (2020). Polyphenols in amaranth (*A. manteggazianus*) flour and protein isolate: Interaction with other components and effect of the gastrointestinal digestion. Food Res. Int..

[53] Dutra R.L.T., Dantas A.M., de Araújo Marques D., Batista J.D.F., de Albuquerque Meireles B.R.L., de Magalhães Cordeiro Â.M.T., Borges G.D.S.C. (2017). Bioaccessibility and antioxidant activity of phenolic compounds in frozen pulps of Brazilian exotic fruits exposed to simulated gastrointestinal conditions. Food Res. Int..

[54] Zhu Y.Q., Zhang A., Tsang D., Huang Y., Chen Z.Y. (1997). Stability of Green tea catechins. J. Agric. Food Chem..

[55] Xiao J. (2017). Dietary flavonoid aglycones and their glycosides: Which show better biological significance?. Crit. Rev. Food Sci. Nutr..

[56] Hollman P.C., Van Trijp J.M., Buysman M.N., vd Gaag M.S., Mengelers M.J., De Vries J.H., Katan M.B. (1997). Relative bioavailability of the antioxidant flavonoid quercetin from various foods in man. FEBS Lett..

[57] Celep E., Charehsaz M., Akyüz S., Acar E.T., Yesilada E. (2015). Effect of *in vitro* gastrointestinal digestion on the bioavailability of phenolic components and the antioxidant potentials of some Turkish fruit wines. Food Res. Int..

[58] Shahidi F., Ambigaipalan P. (2015). Phenolics and polyphenolics in foods, beverages and spices: Antioxidant activity and health effects–A review. J. Funct. Foods.

[59] Kim D.O., Jeong S.W., Lee C.Y. (2003). Antioxidant capacity of phenolic phytochemicals from various cultivars of plums. Food Chem..

[60] Gowd V., Jia Z., Chen W. (2017). Anthocyanins as promising molecules and dietary bioactive components against diabetes–A review of recent advances. Trends Food Sci. Technol..

[61] Poovitha S., Parani M. (2016). *In vitro* and *in vivo* α-amylase and α-glucosidase inhibiting activities of the protein extracts from two varieties of bitter gourd (*Momordica charantia* L.). BMC Complement. Altern. Med..

[62] Tadera K., Minami Y., Takamatsu K., Matsuoka T. (2006). Inhibition of α-glucosidase and α-amylase by flavonoids. J. Nutr. Sci. Vitaminol..

