# Supplementary material for: Effect of Freeze Drying and Simulated Gastrointestinal Digestion on Phenolic Metabolites and Antioxidant Property of the Natal Plum (Carissa macrocarpa)
Source: Foods. 2021 Jun 18;10(6):1420. doi: 10.3390/foods10061420 (PMC8235007; doi:10.3390/foods10061420)
Supplement: Supplementary file 1 [file foods-10-01420-s001.zip › foods-1233223-supplementary.pdf]

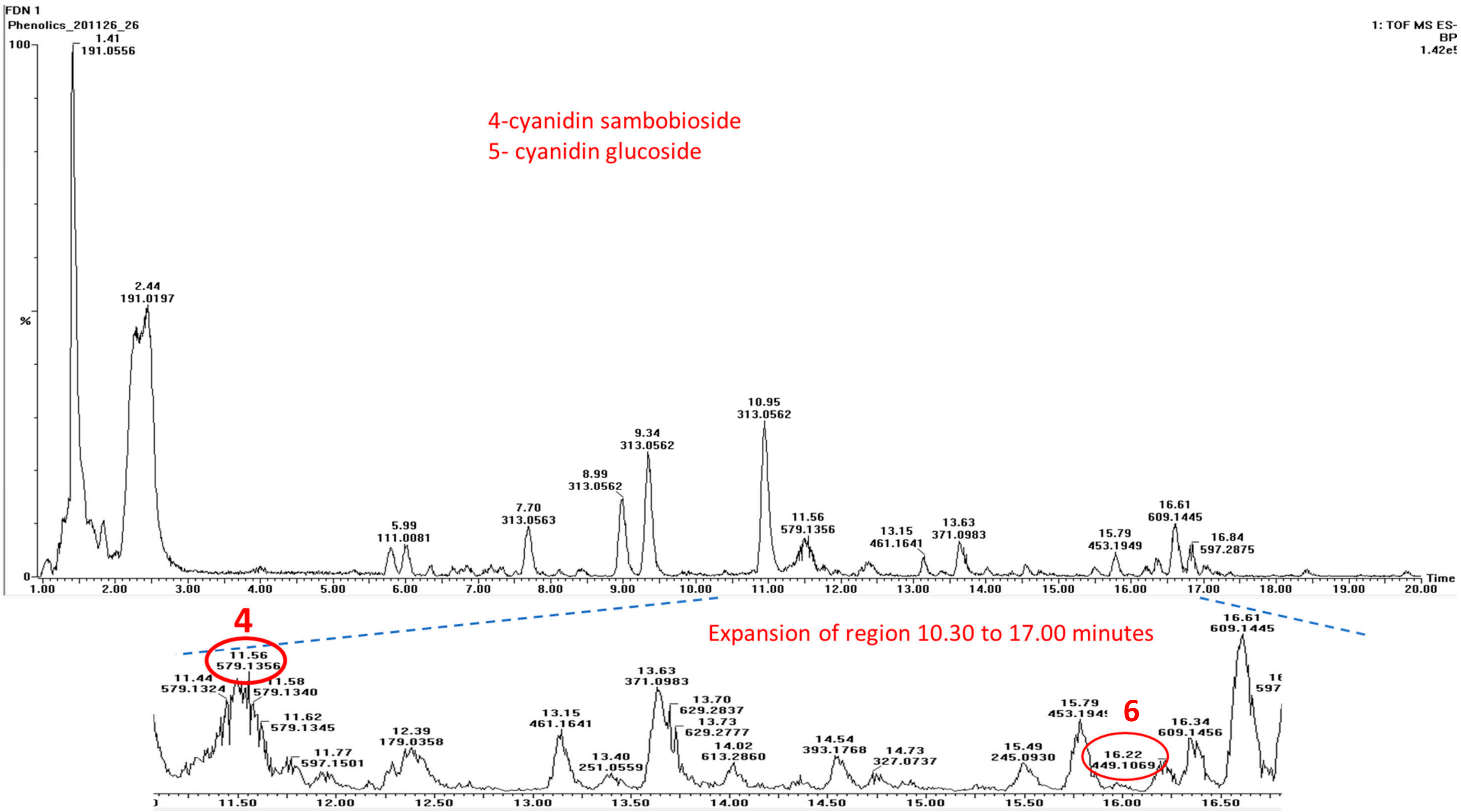

Figure S1 A & B. showing the ESI negative mode BPI chromatogram of the full chromatogram of the hydromethanol extract of Natal plum fruits overlaid with an expansion of the region 10.30 min to 17.00 min of the chromatogram which shows the anthocyanins cyanidin 3-O-β-sambubioside and cyanidin-3-O-

glucoside. Where Figure S1 (A) no 4 shows the *m/z* 579.1356 retention time 11.56 min as cyanidin 3-sambubioside (Cy-3-Sa) and in Figure S1 (B) no 6 at *m/z* 449.1069 retention time 16.22 min was tentatively identified as cyanidin-3-O-glucoside

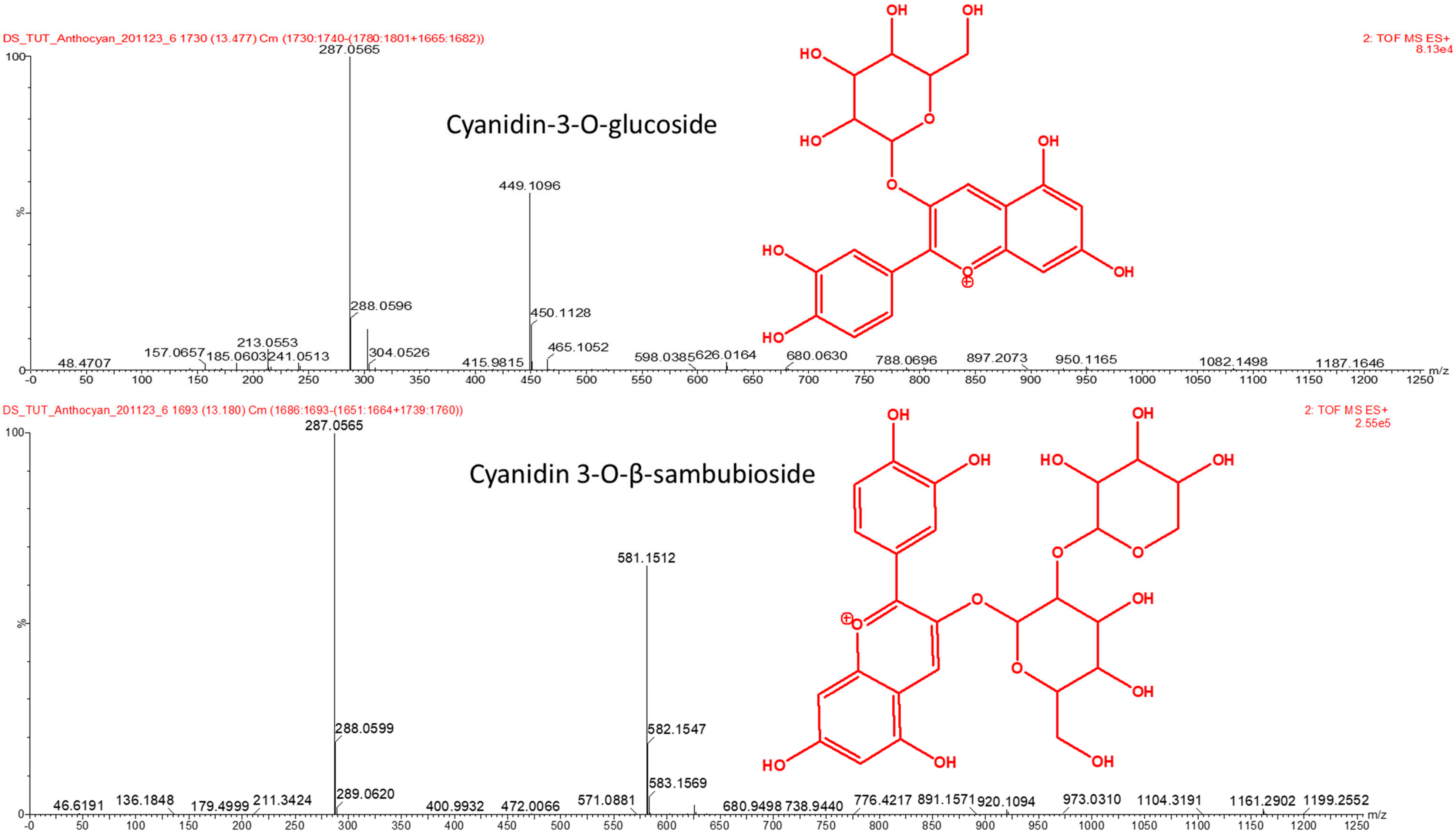

Figure S2A & B shows the MS/MS fragmentation of the identified cyanidin compounds. The Figure S2A shows the Cyanidin-3-O glucoside structure and

MS/MS fragmentation positive mode) overlaid and Figure S 2B shows the Cyanidin 3-O-β-sambubioside and its MS/MS fragmentation pattern in the positive mode

Table S1 Phenolic identification and quantification using HPLC-DAD

Phenolic	Retention time (min)	Regression equation	R^2	LOD (μg/L)	LOQ (μg/L)
Gallic	7.897	y =99324x+55626	0.998	0.05	0.19
Protochatechuic acid	11.255	y=19722x-341718	0.998	3.2	10.9
Catechin	13.24	y=2828x-69172	0.999	3.2	15.7
Epicatechin	14.2	y=35316x+193517	0.997	1.4	3.3
Caffeic	14.509	y=28189x-161653	0.999	2.2	7.2
Chlorogenic	14.724	y=71930x-2110,5	0.995	0.11	0.37
Quercetin	16.77	y=12091x-170181	0.996	7.7	25.8
Ferulic	17. 93	y=20067x-279209	0.999	3.3	11.9
p-Coumaric	17.54	y=6925.3x-62646	0.999	10.25	34.17
Kaempferol	16.448	y=26658x+492185	0.996	2.7	9.3
Syringic	15.44	y=75813x-107617	0.999	0.05	0.18
Ellagic	15. 996	y=20110x+9484.9	0.997	0.31	1.2
Luteolin	24.62	y=27483x+409713	0.998	2.3	7.7

Limit of detection (LOD) and Limit of quantification (LOQ)

Table S2. Tentative peak identification of the compounds detected in the hydromethanol extract of undigested, gastric and intestinal digested Natal plum freeze dried powder

Compound	Retention time (min)	formula	$[M-H]^-$	Error ppm	MSE fragments	Tentative Identification

1	5.56	$C_9H_{17}NO_9$	282.0834	-1.42	211.0031<br>150.0408<br>108.0219	Amino acid derivative
2	8.45	$C_{11}H_{12}N_2O_2$	203.0819	3.45	74.0078<br>116.0517<br>129.0421<br>159.0858	Tryptophan
3	11.48	$C_{21}H_{20}O_{11}$	447.0958	-5.59	285.0359<br>299.0684	Quercitrin
4	11.63	$C_{26}H_{29}O_{15}$	579.1287	11.7	125.0371<br>149.0234<br>147.0614<br>285.0411<br>447.0934	Cyanidin sambubioside
5	15.51	$C_{17}H_{12}NO$	245.0931	-34.7	116.0415<br>129.0372<br>159.0672<br>203.0790	Amino acid derivative
6	16.22	$C_{21}H_{22}O_{11}$	449.1083	1.34	285.0040<br>447.0977	Cyanidin glucoside
7	16.36	$C_{27}H_{30}O_{16}$	609.1399	10.18	300.0276	Quercetin-3-galactoside 7-rhamnoside
8	16.60	$C_{27}H_{30}O_{16}$	609.1445	2.62	179.0415<br>300.0302<br>463.0930	Rutin
9	17.00	$C_{21}H_{20}O_{12}$	463.0977	-20.51	301.0298<br>153.0900	Quercetin-3-galactoside

Table S3 Pearson's correlation coefficient of different phenolic components antioxidant and α-glucosidase activities

Phenolics	r DPPH inhibition IC_{50}	r ABTS inhibition IC_{50}	r FRAP	r α-glucosidase inhibition IC_{50}
Eellagic acid	0.55	0.48	0.25	0.56
Gallic acid	0.68	0.74	0.64	0.66
Protocatechuic acid	0.68	0.59	0.59	0.701
p-Coumaric acid	0.69	0.72	0.83	0.68
Ferulic acid	0.64	0.50	0.45	0.67
Caffeic acid	0.79	0.65	0.55	0.81
Catechin	0.98	0.93	0.85	0.98
Epicatechin	0.97	0.94	0.89	0.98
Kaempferol	0.49	0.49	0.63	0.48
Quercitin	0.69	0.54	0.48	0.71
naringenin	0.66	0.52	0.478	0.70
Apigenin	0.46	0.28	0.27	0.50
Luteolin	0.69	0.55	0.48	0.71
Cyanidin 3-O-sambubioside	0.99	0.95	0.87	0.98
Cyanidin 3-O-glucoside	0.92	0.87	0.85	0.93
Quercitrin	0.76	0.626	0.614	0.79
Quercetin 3 galactoside 7 rhamoside	0.40	0.21	0.20	0.436234
Quercetin 3 galactoside	0.98	0.09	0.256905	0.327719
Quercetin-3-O-rutinoside (Rutin)	0.28	0.98	0.992623	0.93086

Pearson's correlation coefficient =r
